# 2,3,5-Triphenyl Tetrazolium Chloride (TTC): A Dye to Detect Acute Myocardial Infarction in Cases of Sudden Death

**DOI:** 10.7759/cureus.64202

**Published:** 2024-07-09

**Authors:** Ganesh R D, Priyadarshee Pradhan

**Affiliations:** 1 Forensic Medicine, Sri Ramachandra Institute of Higher Education and Research, Chennai, IND

**Keywords:** myocardial infarction, 2-3-5-triphenyl-tetrazolium chloride (ttc), 5-triphenyl-tetrazolium chloride (ttc), sudden death, cardiovascular diseases

## Abstract

Background

Cardiovascular diseases, especially ischemic heart disease, are the most frequent cause of sudden and unexpected death that constitute a significant portion of the autopsies conducted in our country. Though these deaths may be natural as well as unnatural, they carry medico-legal importance because they occur in a person who has been apparently healthy before the supervening of death, and the cause of death is difficult to ascertain. An infarction can be missed by gross and histological examination within the first few hours of sudden death. 2,3,5-triphenyl-tetrazolium chloride (TTC) is a sensitive histochemical method for diagnosing myocardial infarction within four hours of sudden death. The use of such dyes, hence, can possibly aid in ascertaining the cause of death in such cases wherein there are no known preceding factors.

Aim

The aim of this article was to study the occurrence of myocardial ischemia by histochemical staining method - 2,3,5-triphenyl-tetrazolium chloride (TTC).

Methods

This study involved patients who underwent postmortem examination in the Department of Forensic Medicine and Toxicology, Sri Ramachandra Medical College and Research Institute, Chennai.

Results

Of 62 cases, 31 cases were found to be positive for TTC staining, and those heart slices were subjected to histopathological examination. The maximum number of cases (77.4%) showed the age of infarction within zero to four hours, which was detected early by TTC staining compared to microscopic changes in the heart. Only seven cases were positive for myocardial infarction by histopathological examination, proving that it is difficult to detect acute infarction if the age of infarction is less than four hours.

Conclusion

This suggests that for all sudden death cases, 2,3,5-triphenyl tetrazolium chloride could be used as a better tool for the identification of early infarcts.

## Introduction

Death is said to be sudden or unexpected when a person not known to have been suffering from any dangerous disease, injury, or poisoning is found dead or dies within 24 hours after the onset of symptoms. The duration may vary from one hour to 24 hours from the onset of symptoms [1,2].

Sudden and unexpected deaths account for 10%, and the cardiovascular system involves 45% to 50% of sudden deaths. Myocardial infarction due to coronary artery atherosclerosis contributes to approximately 80% of cardiovascular deaths [2].

In 2012, approximately 17.5 million people died from cardiovascular diseases worldwide. This staggering number represented 31% of all global deaths that year. Cardiovascular diseases remain a leading cause of mortality globally, affecting a significant portion of the population across various countries and regions [3].

Cardiovascular disease is a significant cause of death in South Asian countries, including India. It constitutes 24% of deaths in India among the total 53% of non-communicable disease deaths [4,5]. According to a study by Ajay et al., which focuses on South Asians, ischemic heart disease tends to occur five to 10 years earlier in South Asians compared to the Western population. There is a notable gender difference in the age of onset among South Asians with ischemic heart disease. Men tend to experience it at a younger age than women; specifically, South Asian men are approximately 5.6 years younger than South Asian women when encountering ischemic heart disease [6,7]. Cardiovascular diseases, including ischemic heart disease, tend to manifest five to 10 years earlier in India. This means that complications can arise in individuals aged 35-65 years, a demographic crucial to the country's economic productivity. An increase in various risk factors and an unhealthy lifestyle can lead to atherosclerosis, followed by infarction from a younger age. 

Several risk factors contribute to the development of atherosclerosis and subsequent cardiovascular diseases. These include improper dietary habits, excessive smoking, tobacco use, physical inactivity, obesity, alcohol consumption, high levels of cholesterol, increased sugar and blood pressure, socioeconomic factors such as poverty and low educational status, advancing age, and psychological factors [3,7].

Ischemic heart disease can present as clinical syndromes like myocardial infarction, where ischemia causes frank cardiac necrosis; *Angina pectoris* where ischemia is not severe enough to cause infarction, chronic ischemic heart disease with heart failure and sudden cardiac death [8]

Sudden death of cardiovascular origin is frequently associated with coronary artery disease. This emphasizes the significant role of heart-related conditions, such as myocardial infarction, in unexpected deaths.

The diagnosis of myocardial infarction with the postmortem interval presents a different challenge compared to challenges in clinical settings. The confirmation of the diagnosis often requires additional methods beyond gross inspection. During autopsy, biochemical tests using dyes like nitroblue tetrazolium (NBT) and 2,3,5-triphenyl tetrazolium chloride (TTC) are employed to detect areas of myocardial infarction. These dyes help to differentiate regions of normal myocardium from dehydrogenase-deficient myocardium.

In India, the prevalence of cardiovascular-related sudden deaths necessitates a significant number of medico-legal autopsies, and they play a crucial role in determining the cause of death in such cases, particularly in confirming whether myocardial infarction contributed to or caused the death. The aim of the study is to identify the occurrence of myocardial ischemia by histochemical staining method - 2,3,5-triphenyl tetrazolium chloride (TTC) by cross-sectional observational study.

## Materials and methods

In this cross-sectional observational study, a total of 62 cases involving sudden death, road traffic accidents, burns, poisoning, and asphyxia were examined. These cases were brought by police for autopsy to the Forensic Medicine Department of Sri Ramachandra Institute of Higher Education and Research, Chennai, between September 2014 and September 2015. Cases showing signs of decomposition were excluded. Data was expressed in its frequency with percentage and sensitivity, and specificity was derived from true positives, true negatives, false positives, and false negatives.

Before conducting the study, permission was obtained from the Sri Ramachandra University Institutional Research Ethics Committee to use human tissue samples for research purposes. Written consent was also obtained from the blood relatives of the deceased patients after providing a detailed explanation of the study design.

A gross examination of the entire heart was conducted to identify any scars from old myocardial infarctions and areas of softening surrounded by hyperemia.

The preferred method for examining ischemic heart disease was the short-axis method. This involved making transverse cuts approximately 1.0-1.5 cm thick, parallel to the atrioventricular groove, using a long sharp knife [9,10]. Each slice of the heart was examined, starting from the apex and moving towards the base, looking for any signs of ischemia like red-gray infarcts, yellow-tan infarcts, yellow-tan softening, and scarring.

Cold running water was used to briefly wash the heart slices. This step serves to remove clots and excess blood from the surface of the heart tissue. Washing the heart slices helps to prepare them for detailed examination without altering the tissue structure or biochemical composition significantly. The washing process should be brief and not prolonged. Prolonged washing can lead to the elution (washing out) of superficial cellular contents and can potentially alter the appearance of the tissue, providing false readings during subsequent analysis.

Glass jars with a diameter slightly larger than the heart slices were used. Each heart slice was placed in a separate glass jar to ensure individual examination. 2,3,5-triphenyl tetrazolium chloride (TTC) solution was prepared and poured into each glass jar containing the heart slices. The solution level was maintained approximately 2 cm above the heart slices. This depth ensures that the heart slices are fully immersed in the TTC solution. It was imperative to prevent atmospheric oxygen from reaching the heart slices immersed in TTC solution as oxygen can interfere with the chemical reaction that TTC undergoes with dehydrogenase enzymes in viable myocardial tissue.

The incubation of the tissue slices was conducted at room temperature for 60 minutes in a dark room. After 15 minutes of incubation, the tissue slices were turned over to prevent an increased time of contact of the tissue with either the staining solution or the floor of the jar. Prolonged contact could lead to uneven staining or damage to the tissue.

Once the slices showed optimal staining, the incubating medium was poured off from the jars. The jars containing the tissue slices were then filled with a 10% formalin solution, which prevented further enzymatic activity and preserved the cellular structures. This also increased the contrast between the normal and infarcted muscles [11].

## Results

A cross-sectional observational study consisting of 62 cases that were brought for postmortem examination were taken, and of those, myocardium cases were subjected to 2,3,5-triphenyl tetrazolium chloride (TTC). 

Table 1 shows that TTC staining was found to be positive in 31 cases, and those heart slices were subjected to histopathological examination. A maximum number of cases (77.4%) showed the age of infarction within zero to four hours which was detected early by TTC staining compared to microscopic changes in the heart. Only seven cases were positive for myocardial infarction by histopathological examination, as shown in Table 2, proving that it is difficult to detect acute infarction if the age of infarction is less than four hours.

**Table 1:** Survival time and TTC staining TTC - 2,3,5-triphenyl-tetrazolium chloride

Period of survival (age of infarction)	TTC positive (n=31)	Percentage (%)
0-4 hours	24	77.4%
5-8 hours	1	3.2%
8-12 hours	1	3.2%
>12 hours	5	16.2%

**Table 2:** Survival time and H&E staining

Period of survival (age of infarction)	H&E positive (n=7)	Percentage (%)
0-4 hours	0	0.0%
5-8 hours	1	14.28%
8-12 hours	1	14.28%
>12 hours	5	71.42%

Based on these results shown in Table 3, when comparing the TTC stain with the H&E stain, the sensitivity of the TTC stain is 100%. In a larger study population, it can vary from 88.65% to 100%. As one case was false positive, the specificity is 97.5%. In a larger study population, it can vary from 87.12% to 99.56%. 

**Table 3:** Sensitivity and specificity tests ^1^ - one case of false negative because survival time could not be elicited accurately due to improper history from the relatives

Parameter		Estimate	Lower - Upper 95% CIs
Sensitivity		100%	(88.65, 100¹)
Specificity		97.5%	(87.12, 99.56¹)
Positive predictive value		96.77%	(83.81, 99.43¹)
Negative predictive value		100%	(91.03, 100¹)
Diagnostic accuracy		98.57%	(92.34, 99.75¹)
Likelihood ratio of a positive test		40%	(5.634- 284)
Likelihood ratio of a negative test		0.0%	(0.0 - '?')

Table 4 shows the cases of acute myocardial infarction detected by TTC staining and histopathology examination (H&E stain) in various cases.

**Table 4:** Cause of death with TTC and H&E staining TTC - 2,3,5-triphenyl-tetrazolium chloride

Cause Of death	TTC positive (n=31)	H&E positive (n=7)
Sudden death	19	5
Road traffic accident	5	1
Hanging	2	0
Poisoning	3	1
Fall	2	0
Burns	0	0

## Discussion

According to WHO, cardiovascular diseases are the number one cause of death globally [3]. During autopsy, acute myocardial infarction is not detected as death occurs rapidly. Morphological changes of acute myocardial infarction can be visualized grossly between 24 to 48 hours. Usually, diagnosis of myocardial infarction is made by random sectioning of the heart for histopathological examination, where the microscopic changes take four hours to develop [8]. Histopathological examination is of little value if random sectioning of the heart is done, as myocardial infarction can be missed if infarcted area is not included.

The buffered oxidation reduction reagent present in the solution reacts with the normal myocardium and gets stained a brick red color. TTC is used to stain viable myocardial tissue. Healthy, viable tissue containing active dehydrogenase enzymes will metabolize TTC into a red-colored formazan product. In contrast, areas of myocardial infarction or necrosis will not stain red due to the absence of these enzymes. [12].

The study by Adegboyega et al. in 1997 focused on using TTC dye staining in 638 hearts to detect myocardial infarctions from suspected or diagnosed cases. They concluded that for grossly invisible infarctions, TTC is beneficial in the diagnosis of acute myocardial ischemia. The sensitivity of TTC staining for detecting early infarctions was reported to be 77.4%, with a specificity of 92.6%. The overall efficiency of TTC staining for detecting early infarctions was reported as 88% [13]. This is in accordance with the present study because when comparing the TTC stain with the H&E stain, the sensitivity of the TTC stain is 100%. In a larger study population, it can vary from 88.65% to 100%. As 1 case is false positive, the specificity is 97.5%. In a larger study population, it can vary from 87.12% to 99.56%. So, the diagnostic accuracy of 2,3,5 triphenyl tetrazolium chloride in the present study population is 98.57%.

Gupta et al., in 2013, did a study on 100 hearts using H&E as a routine stain and TTC as a gross marker. A positive result with TTC staining was obtained in 68% of cases, while H&E stain showed a positive result in 25% of cases [11].

Kakimoto et al. noted that in cases of sudden death occurring within a few hours after the infarct and without reperfusion, there are minimal histological changes observed in the affected myocardium. In animal models, the earliest time to detect AMI based on dehydrogenase activity as a marker of tissue damage is approximately three hours after the onset of a heart attack. However, human cases require a longer period, specifically at least eight hours [14].

Wachstein and Meisel found the inactivation of enzymes as early as two hours after the onset of acute symptoms [15]. Fishbein et al., in their study, found that using TTC, infarction can be demonstrated within three hours of coronary occlusion [16].

All these studies are in accordance with the present study, where TTC staining was found to be positive in 31 cases. Maximum number of cases (77.4%) showed the age of infarction within zero to four hours, which was detected early by TTC staining in comparison to microscopic changes in the heart. Only seven cases were positive for myocardial infarction by histopathological examination, proving that it is difficult to detect acute infarction if the age of infarction is less than four hours.

Bakkannavar et al., in 2011, concluded that TTC staining demonstrated 100% sensitivity in detecting acute myocardial infarction in 40 hearts from cases of sudden death, suggesting it as a highly effective method [12]. The present study also demonstrates the sensitivity of the TTC stain is 100% when comparing the TTC stain with H&E stain.

TTC detects both acute myocardial infarction as well as old and treated myocardial infarction. So, in cases of sudden death, the cause of death will be acute infarction, while in other unnatural causes of death, a person would have died due to hanging, poisoning, burns, head injury, etc., and infarcts either acute or old would be a contributing factor to the cause of death. From the present study, 19 cases of sudden death were positive for TTC stain while only five cases were H&E positive, proving that when survival time is less than four hours, TTC stain is superior in detecting acute myocardial infarction than H&E stain. While in other unnatural causes of death, acute infarction could have occurred due to a stress response at the time of death, and TTC stain would be positive when survival time is less than four hours in those cases.

The present study demonstrates the use of 2,3,5-triphenyl tetrazolium chloride and the challenges related to variability in survival times, which is often due to difficulties in obtaining accurate historical information from deceased individual's families. This highlights the need for careful documentation and consideration of such factors in post-mortem examination.

The limitations of the study are the information regarding the deceased was based only on the history provided by the police and the relatives making it difficult to calculate the period of survival accurately. Also, the TTC staining method in decomposed bodies may produce false positive results due to autolysis of myocardial tissue. A larger sample size can also be utilized for better understanding of the TTC dye.

## Conclusions

This suggests that for all sudden death cases, 2,3,5-triphenyl tetrazolium chloride could be used as a better tool for the identification of early infarcts. For studying the evolution of ischemic injury of less than four hours, 2,3,5-triphenyl tetrazolium chloride has a greater advantage compared to histopathological examination.

2,3,5-triphenyl tetrazolium chloride (TTC) staining method should be included in the study protocol of all sudden death cases at all institutes throughout the country for diagnosis of myocardial infarction.

## References

[1] Reddy KSN, Murty OP (2014). The Essentials of Forensic Medicine and Toxicology. Medicine and Toxicology, Jaypee Brothers Medical Publishers, 33rd edition.

[2] Aggrawal A (2014). Essentials of Forensic Medicine and Toxicology. 2014.

[3] Federation WH (2011). Global Atlas On Cardiovascular Disease Prevention and Control. https://iris.who.int/handle/10665/44701.

[4] (2011). Noncommunicable diseases country profiles. https://www.who.int/publications/i/item/9789241502283.

[5] (1997). Technical report series. https://extranet.who.int/prequal/vaccines/who-technical-report-series.

[6] (2005). Preventing chronic diseases: a vital investment. https://www.ncdseminar.org/archive/00_fichiers/Docs/WHO/Prevention-ncd-vital-invest-overview%28WHO-2005%29.pdf.

[7] Ajay VS, Prabhakaran D (2010). Coronary heart disease in Indians: Implications of the INTERHEART study. Indian J Med Res.

[8] Kumar V, Abbas AK, Aster JC (2015). Robbins and Cotran Pathologic Basis of Disease. https://shop.elsevier.com/books/robbins-and-cotran-pathologic-basis-of-disease/kumar/978-0-323-53113-9.

[9] Ludwig J (2002). Handbook of Autopsy Practice. Cardiovascular system.

[10] Kotabagi RB, Apte VV, Pathak PR (2000). Post mortem diagnosis of early myocardial infarction. Med J Armed Forces India.

[11] Gupta B, Gohel H, Desai N, Dodhia S (2024). Post mortem study of heart in cases of sudden cardiac death using triphenyl tetrazolium chloride and Haematoxylin & Eosin stain. Indian J Appl-Basic Med Sci.

[12] Shenoy RP, Bakkannavar SM, Monappa V (2011). Identification of myocardial Infarction in human autopsy population using TTC. J Pharm Biomed Sci.

[13] Adegboyega PA, Adesokan A, Haque AK, Boor PJ (1997). Sensitivity and specificity of triphenyl tetrazolium chloride in the gross diagnosis of acute myocardial infarcts. Arch Pathol Lab Med.

[14] Kakimoto Y, Tsuruyama T, Miyao M (2013). The effectiveness and limitations of triphenyltetrazolium chloride to detect acute myocardial infarction at forensic autopsy. Am J Forensic Med Pathol.

[15] Wachstein M, Meisel E (1955). Succinic dehydrogenase activity in myocardial infarction and in induced myocardial necrosis. Am J Pathol.

[16] Fishbein Fishbein, Meerbaum Meerbaum (1981). Early phase acute myocardial infarct size quantification: validation of the triphenyl tetrazolium chloride tissue enzyme staining technique. Am Heart J.

